# Lipid profiles of prostate cancer cells

**DOI:** 10.18632/oncotarget.26222

**Published:** 2018-10-30

**Authors:** Alexandra Sorvina, Christie A. Bader, Chiara Caporale, Elizabeth A. Carter, Ian R.D. Johnson, Emma J. Parkinson-Lawrence, Peter V. Simpson, Phillip J. Wright, Stefano Stagni, Peter A. Lay, Massimiliano Massi, Douglas A. Brooks, Sally E. Plush

**Affiliations:** ^1^ Mechanisms in Cell Biology and Disease Research Group, School of Pharmacy and Medical Sciences, Sansom Institute for Health Research, University of South Australia, Adelaide, Australia; ^2^ School of Molecular and Life Science – Curtin Institute for Functional Molecules and Interfaces, Curtin University, Bentley, Australia; ^3^ Sydney Analytical and School of Chemistry, The University of Sydney, Sydney, Australia; ^4^ Department of Industrial Chemistry “Toso Montanari”, University of Bologna, Bologna, Bologna, Italy; ^5^ Future Industries Institute, University of South Australia, Mawson Lakes, Australia

**Keywords:** prostate cancer, lipid profiles, LC-ESI-MS/MS, FTIR, lipid dyes

## Abstract

Lipids are important cellular components which can be significantly altered in a range of disease states including prostate cancer. Here, a unique systematic approach has been used to define lipid profiles of prostate cancer cell lines, using quantitative mass spectrometry (LC-ESI-MS/MS), FTIR spectroscopy and fluorescent microscopy. All three approaches identified significant difference in the lipid profiles of the three prostate cancer cell lines (DU145, LNCaP and 22RV1) and one non-malignant cell line (PNT1a). Specific lipid classes and species, such as phospholipids (e.g., phosphatidylethanolamine 18:1/16:0 and 18:1/18:1) and cholesteryl esters, detected by LC-ESI-MS/MS, allowed statistical separation of all four prostate cell lines. Lipid mapping by FTIR revealed that variations in these lipid classes could also be detected at a single cell level, however further investigation into this approach would be needed to generate large enough data sets for quantitation. Visualisation by fluorescence microscopy showed striking variations that could be observed in lipid staining patterns between cell lines allowing visual separation of cell lines. In particular, polar lipid staining by a fluorescent marker was observed to increase significantly in prostate cancer lines cells, when compared to PNT1a cells, which was consistent with lipid quantitation by LC-ESI-MS/MS and FTIR spectroscopy. Thus, multiple technologies can be employed to either quantify or visualise changes in lipid composition, and moreover specific lipid profiles could be used to detect and phenotype prostate cancer cells.

## INTRODUCTION

A hallmark of cancer cells is their metabolic reprogramming, which enables them to rapidly proliferate, migrate and alter their microenvironment to facilitate metastasis. Characteristics of metabolic alterations in cancer cells include significant increases in glucose and glutamine consumption [1], and alterations in lipid transport and utilisation [2]. Unlike many other cancer cells, prostate cancer cells exhibit a major reliance upon the uptake and metabolism of fatty acids, when compared to glucose uptake and glycolysis [3]. Overexpression of fatty acid synthase (FAS) is associated with an increased risk of mortality from prostate cancer, which rises further when combined with a loss of the phosphatase and tensin homolog (PTEN) tumour suppressor gene [4]. The loss or inactivation of PTEN induces an upregulation of FAS that is mediated via the activation of phosphoinositide 3-kinase (PI3K)/AKT pathway [5]. Furthermore, concomitant activation of PI3K/AKT and MAPK pathways increases sterol regulatory element-binding protein (SREBP)-dependent lipogenesis [6]; compounding the downstream effects of androgen-regulated (AR) metabolism [7], lipid composition and architecture of cellular membranes. The altered synthesis and metabolism of lipids in prostate cancer cells and changes to lipid profiles is therefore being recognised as a key feature of the pathogenesis [8, 9].

Altered lipid signalling pathways and lipid signatures may offer insights into the metabolic reprograming that occurs in prostate cancer cells and disease progression. Thus, the quantitation and visualisation of the cellular lipids may aid in the understanding of prostate cancer pathogenesis. Lipidomics of prostate cancer using mass spectrometry (MS) has already shown that cholesteryl esters (CE) are present at higher concentrations than in a normal prostate tissue [10]. Moreover, significant alterations have been observed in prostate cancer patient plasma concentrations of phosphoethanolamine (PE), ether-linked PE, phosphatidylinositol, ether-linked phosphatidylcholine (PC), sphingomyelin (SM) and ceramide [8, 11]. Alternative technologies, such as Fourier transform infrared spectroscopy (FTIR), have also been applied to identify changes in prostate cancer tissues in an effort to identify biomarkers for diagnosis [12, 13]. Changes in lipid features could be identified in prostate cancer patient samples and were correlated with patient grading [12]. Although this approach is only able to identify lipids down to a class level, FTIR does have a number of advantages over MS, as it does not require internal standards, can be applied to intact cells or tissue samples for lipid profiling and can provide spatial as well as quantitative information [14]. Fluorescence microscopy is another approach which can provide important spatial information about lipids with minimal requirements for sample preparation. To date this approach has been limited by the number of fluorescent stains available for the detection of endogenous lipids. Several lipophilic dyes such as Oil Red O, Nile Red and BODIPY^®^ 493/503 have been available for some years; however, these dyes primarily localise with neutral lipids, such as CE and triacylglycerides (TAG), and not with polar lipids, which have been implicated in prostate cancer pathogenesis from studies on plasma lipodomics. The availability of next generation dyes such as, the luminescent Rhenium(I) complex, ReZolve-L1™, which has been shown to localise in areas of high polar lipid content (e.g. in close association with SM, PE and PC) in live and fixed adipocytes [15], warrant the investigation of fluorescence imaging as a potential method in lipid profiling.

Although lipid signatures have been identified in primary tumour tissues and plasma samples from prostate cancer patients, there has yet to be a study evaluating lipid profiles within cell models of prostate cancer. Therefore, in this study several approaches were utilized including liquid chromatography-electrospray ionization-tandem mass spectrometry (LC-ESI-MS/MS), FTIR spectroscopy and fluorescence imaging to characterise lipid profiles in a number of prostate cells lines. Three prostate cancer cell lines DU145, 22RV1 and LNCaP and one non-malignant PNT1a cells were used to compare lipid profiles. DU145 represents late stage prostate cancer with moderate metastatic potential [16], and is neither hormone-sensitive nor expresses PSA. The cell lines, 22RV1 and LNCaP more closely mimic aspects of the more common clinical disease, as they are androgen-responsive and express PSA [17]. The lipid profiles obtained indicated that these cell lines provide comparable lipid changes to those reported in patient samples and further demonstrate that multiple approaches can be used to provide insight into lipid content of cells.

## RESULTS

**Figure 1:**
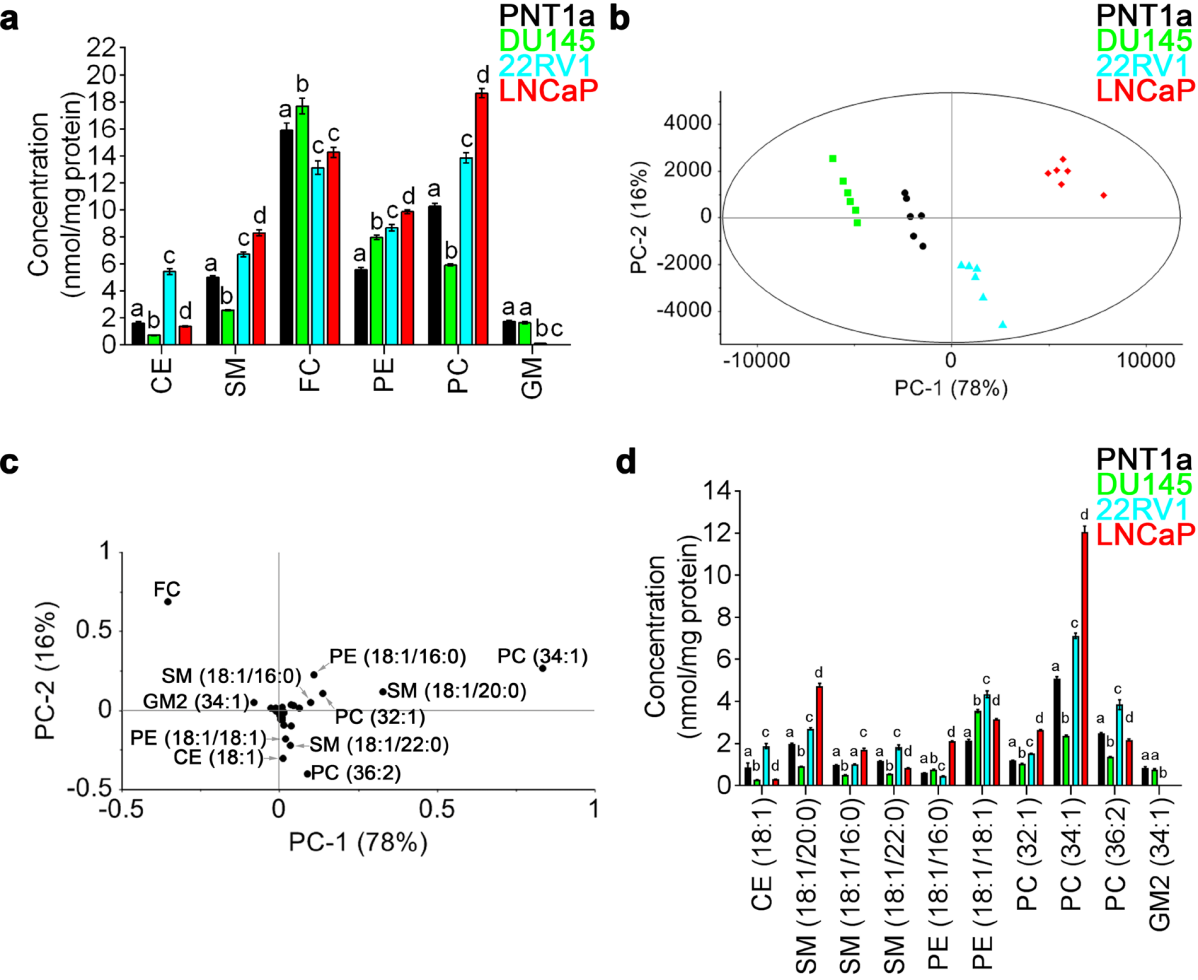
Quantitative LC-ESI-MS/MS data analysis of the relative abundances of lipids in prostate cell lines **(a)** Comparison of average concentrations [nmol mg^-1^ protein] of (CE) cholesteryl esters, (SM) sphingomyelin, (FC) free cholesterol, (PE) phosphatidylethanolamine, (PC) phosphatidylcholine and (GM) gangliosides in prostate cell lines for six parallel samples with their standard error. One-way ANOVA and Tukey's multiple comparison tests showed significant differences among the means for the samples (depicted by different letters on the bars, *p* < 0.05). **(b)** The PCA scores plot comparing non-malignant PNT1a (black circles) and prostate cancer cell lines DU145 (green squares), 22RV1 (blue triangles) and LNCaP (red diamonds), using identified lipid species. **(c)** Loadings plot of PCA for PC-1 (equal to 78%). **(d)** Comparison of average concentrations [nmol mg^-1^ protein] of lipids that allowed the differentiation of non-malignant PNT1a and prostate cancer cell lines, DU145, 22RV1 and LNCaP (*p* < 0.05). Data presented as mean ± SEM of six independent biological replicates for each of the four prostate cell lines.

LC-ESI-MS/MS was performed on total lipid extracts prepared from non-malignant PNT1a and three prostate cancer cell lines, DU145, 22RV1 and LNCaP. A total of 53 lipid species were quantified, including 30 detected in positive-ion mode and 23 detected in negative-ion mode; covering six different lipid classes, including CE, SM, free cholesterol (FC) and PC in positive-ion mode, and PE and gangliosides (GM) in negative-ion mode (Supplementary Table 1). PE showed increased levels in all three prostate cancer cell lines, when compared to non-malignant PNT1a cells (Figure 1a). An increase in SM and PC was detected in 22RV1 and LNCaP cells, when compared to PNT1a cells, while DU145 cells displayed significantly reduced amounts of these lipids (Figure 1a). The concentration of CE, FC and GM varied between cell lines, and there was no consistent change in these lipids that distinguished prostate cancer derived cell lines from non-malignant PNT1a cells (Figure 1a).

To further interrogate the difference in lipid profiles between the four cell lines under investigation, principal component analysis (PCA) was performed on all 53 lipid species. In this analysis, prostate cancer cells lines were visually separated from one another and from the non-malignant cell line PNT1a along the PC-1 and PC-2 axes, which accounted for 78% and 16% of the overall variance in the data, respectively (Figure 1b). The scores plot showed distinct separation of each prostate cell line along PC-1, with DU145 cells exhibiting the most negative scores and LNCaP cells exhibiting the most positive scores. PNT1a and 22RV1 cells were also observed to separate along the PC-1 axis, but their location close to the centre indicates less variability exists between these cell lines (Figure 1b). The separation of PNT1a from LNCaP cells was much greater than for either 22RV1 or DU145 prostate cancer cells. The PC-1 and PC-2 loadings plot suggested that 11 lipid species accounted for the principal differences between the four cell lines; FC, CE (18:1), PE (18:1/16:0), PE (18:1/18:1), PC (32:1), PC (34:1), PC (36:2), SM (18:1/20:0), SM (18:1/16:0), SM (18:1/22:0) and GM2 (34:1) (Figure 1c). The lipid species that were located close to zero on the loadings plot had minimal capacity for differentiating between cells lines (Figure 1c). By comparing the loadings plot with the scores plot, it was evident that lipid profiles for LNCaP cells are dominated by PE (18:1/16:0), PC (32:1), PC (34:1), SM (18:1/20:0) and SM (18:1/16:0) (Figure 1b, 1c). In 22RV1 cells, CE (18:1), PE (18:1/18:1), PC (36:2) and SM (18:1/22:0) were the dominant lipid species (Figure 1b, 1c). In DU145 cells, FC was the most abundant (Figure 1d) and the most dominant lipid species (Figure 1b, 1c). Of the lipids identified by PCA, PE (18:1/18:1) was the only lipid species that showed increased abundance across all three prostate cancer cell lines, when compared to PNT1a cells (Figure 1d). Direct comparison of each prostate cancer cell line with PNT1a is illustrated as a volcano plot (Supplementary Figure 1), which was generated based on the fold change (where 1 indicates no change) and *p* value (*p* < 0.05) for a *t*-test of differences between PNT1a and prostate cancer cell lines (i. e., DU145, 22RV1 and LNCaP). This analysis also identified the 11 lipid species identified by PCA analysis to be significantly different between PNT1a and both 22RV1 and LNCaP prostate cancer cell lines. In addition it showed a significant increase in the level of PE (18:1/18:0) in all three cancer cell lines compared to PNT1a and significant change in CE (18:1) for all three. Some of the less abundant lipid species, which were not identified as significant contributors to the separations in the PCA, where also identified by this approach when compared to PNT1a cells, for example, unsaturated CE species, CE (20:3) and CE (22:4) were at least twice as abundant in prostate cancer cells, when compared to PNT1a cells (Supplementary Figure 1, Supplementary Table 1). Thus, the abundance of specific SM and PC species, along with CE, may assist in differentiating prostate cancer cells. Significantly lipid profiles varied between prostate cancer cell lines, an observation which reflects the heterogeneous nature of this disease, but which may provide a useful tool for the phenotyping of prostate cancer.

**Figure 2:**
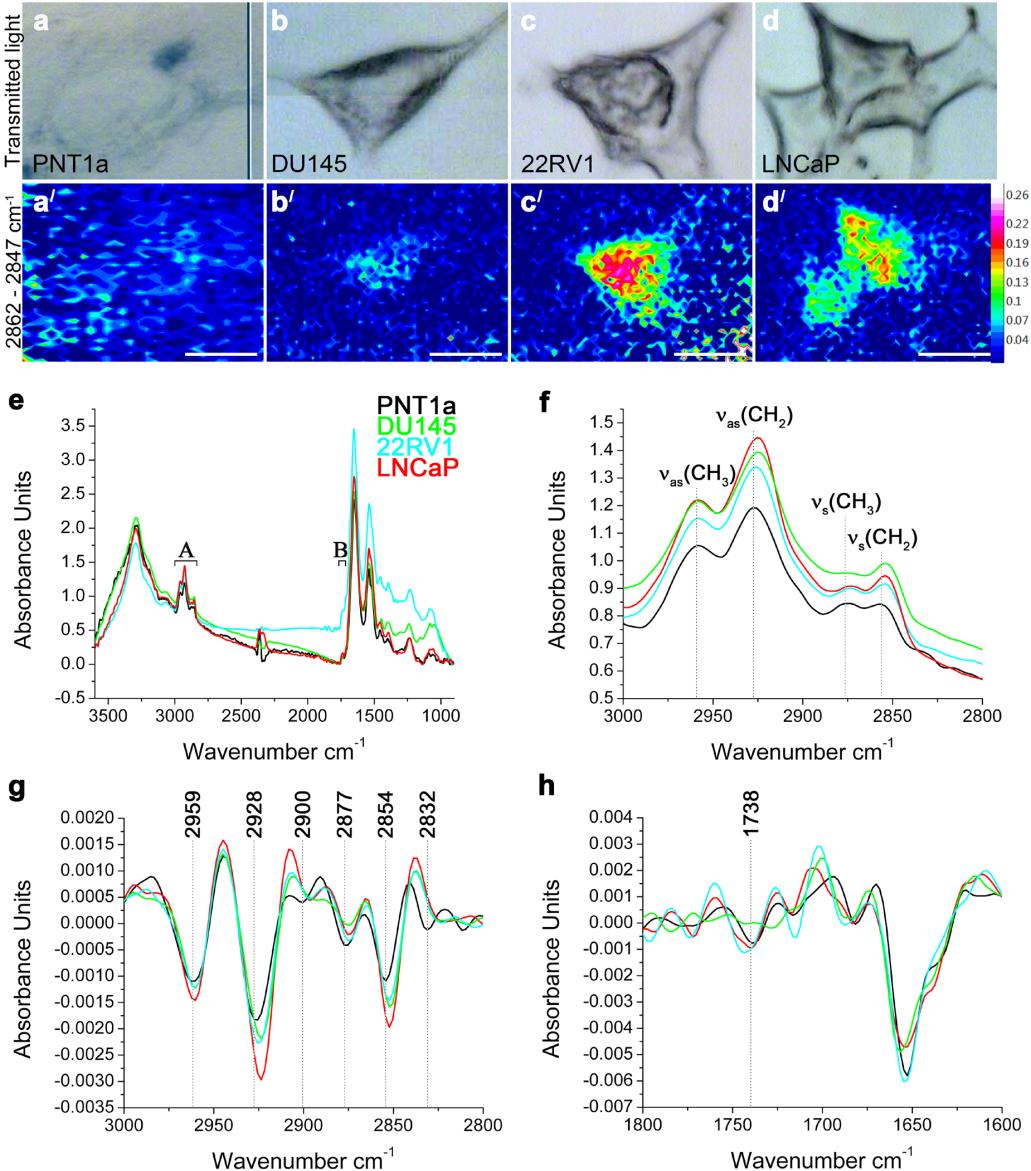
FTIR spectroscopy on prostate cancer cells **(a-d)** Optical and **(a**^**/**^**-d**^**/**^**)** FTIR images collected by integrating the area under the ν_s_(CH_2_) band (2862–2847 cm^-1^) and generated from (a, a^/^) PNT1a and prostate cancer cell lines, (b, b^/^) DU145, (c, c^/^) 22RV1 and (d, d^/^) LNCaP. **(e)** Average FTIR spectra from PNT1a (black) and prostate cancer cell lines, DU145 (green), 22RV1 (blue) and LNCaP (red), acquired over the 3600–900 cm^-1^ spectral region, where (A) C–H and (B) C=O bands were located. **(f)** Averaged spectra of the C–H stretching region (3000–2800 cm^-1^) with band assignment. **(g)** Second-derivative spectra of the C–H stretching region shown in f. **(h)** Second-derivative spectra of the C=O stretching region.

To illustrate the lipid distribution and relative abundance within cells of these four cell lines, FTIR spectroscopy was used to map lipids in individual cells (Figure 2). The FTIR spectroscopy can recognise lipids to a class-level, does not require internal standards and can be applied to intact cells to generate a molecular image of the sample without the need for prior target identification [12, 14, 18]. The requirement of this technique is the use of samples of an appropriate thickness to allow a sufficiently large absorbance intensity to be recorded from the cell and to avoid recordings of spectra from the underlying substrate. Although the thickness of prostate cells varied making consistent sampling across individual cells difficult and resulting in low signal-to-noise ratio, an initial insight into the lipid biology of these cells was obtained using FTIR spectroscopy. Spectra were acquired over 3600–900 cm^-1^ range, where C–H (∼3000–2800 cm^-1^) and C=O (∼1750–1700 cm^-1^) bands were located. The low signal to noise made mapping individual lipid groups within the cells difficult, however FTIR images of total lipid content were generated by integrating the area under the ν_s_(CH_2_) band (2862–2847 cm^-1^). This demonstrated that the lipids were not homogeneously distributed within the prostate cells; with the highest intensity observed in the perinuclear region and the lowest intensity at the cell periphery (Figure 2a^/^-2d^/^). Prostate cancer cell lines 22RV1 and LNCaP cells had higher lipid concentrations (indicated by the higher intensity) and wider distribution of lipids, when compared to DU145 and PNT1a (Figure 2a^/^-2d^/^) cells. Analysis of the FTIR spectra collected from prostate cancer cell lines confirmed the trend of increased intensity of lipid ν(CH) bands (Figure 2e, 2f). In addition, the position of the ν_as_(CH_2_) and ν_s_(CH_2_) bands shifted in the spectra of all three prostate cancer cells, when compared to PNT1a cells, which suggests altered lipid composition (Supplementary Table 2). PCA was also performed to interrogate two spectral regions (i. e. 3000–2800 cm^-1^ and 1750–1700 cm^-1^; Figure 2g, 2h) containing lipid vibrational modes across the spectra collected from each cell line. In the scores plots (Supplementary Figure 2a, 2c), PNT1a and prostate cancer cell lines, 22RV1 and LNCaP, had a better spatial separation along the PC-1 axis than with DU145 cells. Given the small sample size, the PCA analysis revealed poor separation of the cell lines on the plots. The PCA plot displayed positive loadings in the region of 2900–2880 cm^-1^ and 2840–2830 cm^-1^ (Supplementary Figure 2b) associated with C-H stretching modes and the 1750–1734 cm^-1^ region (Supplementary Figure 2d) associated with the lipid ν(C=O) bands. Reference spectra, obtained for a range of pure lipid standards, indicated that peaks in the C-H stretching region were likely to be associated with FC or CE, while peaks in the C=O stretching corresponded with those of PE, PC and CE (Supplementary Table 3). These findings, in combination with the increase in the intensity of the ν(C-H) and ν(C=O) bands, indicate that prostate cancer cell lines exhibit altered lipid composition, which was detectable in individual cells and is in agreement with the LC-ESI-MS/MS findings on cell extracts.

**Figure 3:**
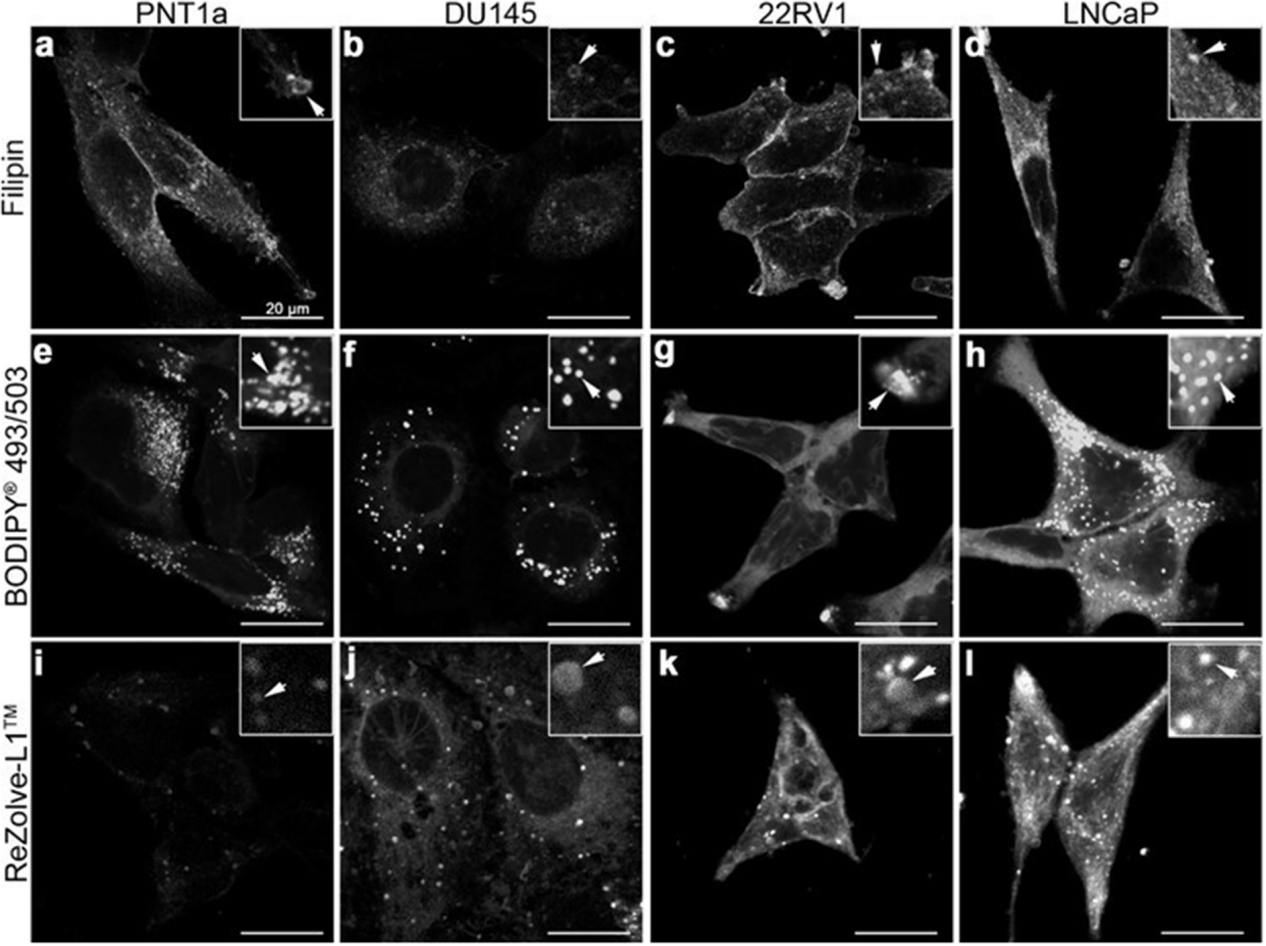
Distribution of lipids in prostate cancer cells **(a-l)** Micrographs of cross-sections through prostate cells that show the intracellular location of neutral and polar lipids. Cholesterol was depicted by staining cells with Filipin III (a-d). Neutral lipids such as triglycerides and cholesteryl esters were detected by staining cells with BODIPY^®^ 493/503 (e-h). ReZolve-L1™ (i-l) was used for staining polar lipids. Representative images from non-malignant PNT1a (a, e, i) and prostate cancer DU145 (b, f, j), 22RV1 (c, g, k) and LNCaP (d, h, l) cell lines. Prostate cells were fixed with 4% PFA (a-h) or imaged live (i-l). Scale bars, 20 μm.

To better determine lipi d localisation at a cellular level fluorescence microscopy was performed using a range of lipid localising dyes. Three commercially available lipid dyes were utilised, Filipin III was used for the visualisation of FC, BODIPY^®^ 493/503 was selected for the detection of CE and TAG in lipid droplets and ReZolve-L1™ was chosen for its localisation with polar lipids, such as SM and PE [15, 19, 20]. Filipin III staining for FC revealed numerous intracellular vesicles in all four cells lines, with the brightest intracellular staining detected in LNCaP cells, followed by PNT1a cells (Figure 3a-3d). Cholesterol was detected in the region of the plasma membrane by Filipin III in PNT1a, LNCaP and 22RV1 cells and was particularly intense in 22RV1 cells (Figure 3a, 3c, 3d). In contrast, the fluorescent signal from Filipin III staining in DU145 cells was minimal and restricted to the cytoplasm with no apparent plasma membrane staining. BODIPY^®^ 493/503 detected distinctive punctate structures throughout cells consistent with lipid droplets, where CE and TAG are known to reside. These compartments were distributed throughout the cytoplasm in PNT1a, DU145 and LNCaP cells (Figure 3e, 3g, 3h), but were accumulated in the cellular projections of 22RV1 cells (Figure 3g). BODIPY^®^ 493/503 detected many small lipid droplets in PNT1a cells (Figure 3e), while droplets in DU145 and LNCaP were comparatively large (Figure 3f, 3h). In the case of DU145 fewer (Figure 3f) lipid droplets were observed. In both 22RV1 and LNCaP cells, BODIPY^®^ 493/503 was detected as diffuse cytosolic staining (Figure 3g, 3h), suggesting increased lipophilicity in the cytoplasm, which was not seen in the other two cell lines (Figure 3e, 3f). In all three prostate cancer cell lines, polar lipid detection by ReZolve-L1™ detected small punctate structures, larger vesicular structures and extensive lipid networks throughout the cytosol, whereas in PNT1a cells ReZolve-L1™ demonstrated only weak staining of punctate structures (Figure 3i-3l). The phosphorescence intensity from ReZolve-L1™ in PNT1a cells was only minimally above the detection threshold, which was in stark contrast to DU145, 22RV1 and LNCaP cells, which had intense emission patterns (Figure 3i-3l, Supplementary Figure 3e; *p* < 0.05). Quantification of emission intensity confirmed that DU145, LNCaP and 22RV1 cells had significantly greater ReZolve-L1™ staining when compared to PNT1a cells (Supplementary Figure 3). This demonstrated that fluorescence imaging can be utilised to detect changes in lipid content and distribution within prostate cell lines, directly complementing the information obtained from LC-ESI-MS/MS and FTIR spectroscopy.

## DISCUSSION

The heterogeneous nature of prostate cancer was reflected in the lipodomics analysis with significant variations reported between prostate cancer cells lines as well as between the non-malignant PNT1a cells and cancer cell lines. The key lipid classes identified in the AR-positive cell lines, LNCaP and 22RV1, were overlapping with the three-lipid signatures (i.e. SM, ceramide and PC) found in metastatic castration-resistant prostate cancer plasma samples [11]. The AR-negative cell line, DU145, showed a significantly altered lipid profile in relation to PNT1a cells, and this relationship appeared to be the inverse of LNCaP and 22RV1 cells.

Differences observed in lipid profiles of prostate cells lines by LC-ESI-MS/MS, were also able to be detected at an individual cell level by FTIR spectroscopy and by fluorescent markers. However, both FTIR and fluorescence microscopy were limited in their resolution of lipid species and quantitative ability. Interestingly, the LC-ESI-MS/MS revealed elevated levels of FC in DU145 cells, but Filipin III staining of FC showed minimal visualisation in these cells. The increased staining of FC in LNCaP and 22RV1 cells, which was consistent with previous observations [21], did not match expected outcomes based on LC-ESI-MS/MS results. Filipin III staining can be altered through cross-linking and mobilisation from aldehyde-based cell fixation methods [22]. In addition, Filipin III is known to display a nonlinear fluorescence response to FC abundance, which makes it unsuitable for accurate quantitation [23]. Like FC, accumulated CE has also been shown in prostate cancer cells [9], consistent with this neutral lipid accumulation detected by BODIPY^®^ 493/503 fluorescence; with increased cytoplasmic staining observed in 22RV1 and LNCaP cells and increased lipid droplet size in DU145 cells, when compared to PNT1a. Although the LC-ESI-MS/MS data indicated the highest abundance of CEs was in 22RV1 cells, DU145 and LNCaP cells both had significantly lower total CE than PNT1a. This was contrary to FTIR data, which indicated that LNCaP cells had only a slight reduction in the intensity of the band associated with ester groups, compared to 22RV1 cells. This could relate to the localisation of lipid accumulation in 22RV1 cells as staining with BODIPY^®^ 493/503 showed lipid accumulation in the cell periphery where detection by FTIR was limited by sample thickness. Furthermore, using FTIR and BODIPY^®^ 493/503 it is not possible to differentiate between CE and TAG therefore it is possible that the observed changes may relate to accumulation of other neutral lipids such as fatty acids and TAGs that have not been analysed in this study. These findings demonstrate a current limitation of fluorescence microscopy (due to the lack of availability of specific dyes) and FTIR, particularly for accurate assessment of cellular cholesterol species and demonstrate the need for careful consideration of the tools available when preforming lipid analysis.

Increased levels of all classes of phospholipids analysed were observed in prostate cancer cells compared to PNT1a using all analytical methods. Detailed analysis by LC-ESI-MS/MS analysis, found PE (18:1/18:1) was one of the few lipid species to be enriched in all three prostate cancer cell lines, when compared to PNT1a cells. Similarly, PE (18:1/18:1) has been detected in high abundance in exosomes derived from PC-3 cells [24] and in prostate cancer patient-derived plasma samples [8, 25]. Correspondingly, exosomes from patient urine have lipid signature characterised by increased PE, which were detected as ether-linked PE species [25]. Cellular PE can be converted to PC by phosphatidylethanolamine *N*-methyltransferase [26], and when compared to PNT1a, the PC content was increased in 22RV1 and LNCaP cells, but was reduced in DU145 cells. Two species of this lipid class, PC (32:1) and PC (36:2), were elevated in 22RV1 and LNCaP prostate cancer cells. This observation was consistent with increases in these lipid species in other cancers [27–29] and with PC (34:1), which has also been reported to be increased in prostate cancer [8]. Differential abundance of saturated phospholipids, such as PE, PC and phosphatidylserine, markedly alter signal transduction and can protect cancer cells from oxidative stress-induced cell death [30, 31]. These phospholipids have been directly linked to cancer cell proliferation, involving Akt mediated signalling interactions between Raf-1 kinase inhibitory protein (RKIP) and PE [32], and the subsequent modulation of ROS production [33, 34].

Bioactive sphingolipids, such as ceramide and sphingosine, act as effector molecules in cell signalling and can regulate the response of prostate cancer cells to chemotherapy or radiation [35]. An increase in ceramides in cancer cells can be achieved through the hydrolysis of SM [35]. Similarly to PC, the amounts of SM were increased in 22RV1 and LNCaP cells, but not in DU145 cells, when compared to PNT1a cells. The increased levels of SM (18:1/20:0) in 22RV1 and LNCaP may also relate to the altered release of exosomes from prostate cancer cells [36]. Similar to PC-3 prostate cancer cells [36], 22RV1 and LNCaP displayed low amounts of GMs (e. g., GM2 (34:1)). Although GMs are potential biomarkers for lung [37] and breast [38] cancers, the exact species that contribute to cancer progression have yet to be identified. As sphingolipids are implicated in the regulation of steroidogenesis [39], the differential lipid profile observed in 22RV1 and LNCaP cells, compared with both DU145 and PNT1a cells, may reflect their AR status. Androgens markedly influence the synthesis and uptake of fatty acids in prostate cells, and proteins involved in lipid metabolism may be influenced by differential modulation of the AR [40]. SM, ceramide and PC have been used as a three-lipid signature to identify poor prognostic outcomes in castration-resistant prostate cancer [11]. This aligning of castration-resistant outcomes and the observations in cancer cells suggests that the identification of specific lipid signatures may aid in identifying novel biomarkers for prostate cancer.

The lipid profiles obtained from LC-ESI-MS/MS and FTIR spectroscopy were good predictors for the subsequent staining of polar lipids with ReZolve-L1™. The LC-ESI-MS/MS analysis of prostate cancer 22RV1 and LNCaP cells demonstrated elevated amounts of PE, PC and SM, which have previously demonstrated an association with ReZolve-L1™ [20]. These lipids have structural and functional consequences for cancer cell pathogenesis and disease progression. However, the correlation between intensity values of ReZolve-L1™ and levels of certain lipid classes are yet to be established. The visualisation of the altered polar lipids with ReZolve-L1™ and apparently different patterns of lipid location within each of the prostate cancer cells suggested that lipid handling and metabolism may have the capacity to identify each of the cancer cell lines as a unique visual entity.

In summary, our work has provided further evidence of the role that lipids play in prostate disease, the need to carefully align cell choice when mapping in cellulo studies with those in tissue and to highlight the suitability of three analytical methods to the analysis of lipid profiles of cell models.

## MATERIALS AND METHODS

### Cell lines and culture conditions

Human prostate non-malignant PNT1a and prostate cancer 22RV1, LNCaP (clone FCG) and DU145 cell lines were obtained from the European Collection of Cell Cultures via CellBank Australia (Children's Medical Research Institute, Westmead, NSW, Australia). PNT1a and 22RV1 cells were maintained in RPMI-1640 medium (#R0883, Sigma-Aldrich, USA), supplemented with 10% fetal bovine serum (#IVT3008403, *In Vitro* Technologies, Australia) and 2 mM L-glutamine (#25030-081, Gibco^®^, USA). LNCaP cells were maintained in RPMI-1640 medium, supplemented with 2 mM L-glutamine, 10 mM HEPES (#H0887, Sigma-Aldrich, USA) and 1 mM sodium pyruvate (#S8636, Sigma-Aldrich, USA). DU145 cells were cultured in MEM medium (#M5650, Sigma-Aldrich, USA), supplemented with 10% fetal bovine serum, 2 mM L-glutamine and 1 mM sodium pyruvate. The prostate cell lines were incubated at 37°C with 5% CO_2_ in a Sanyo MCO-17AI humidified incubator (Sanyo Electric Biomedical Co., Ltd.). Cells were passaged at approximately 80% confluence, for detachment TrypLE™ Express (#12604-021, Gibco^®^, USA) was used. The PNT1a cell line was chosen as a non-malignant control for comparison to three cancer cell lines, 22RV1, LNCaP and DU145. Prostate cancer lines 22RV1 and LNCaP closely mimic aspects of the more common clinical disease, as they are AR-responsive and express PSA [17]. On the other hand, DU145 represents a late-stage prostate cancer with moderate metastatic potential [16, 41], and is neither hormone-sensitive nor expresses PSA.

### Lipid extraction and LC-ESI-MS/MS analysis

Prostate cell lines were seeded in 75 mm^2^ flasks (*n* = 6 for each cell line) at 1 x 10^5^ cells mL^−1^. At ∼80% confluence, cells were harvested and cell pellets were stored at -80°C until required for lysis. For lysis, each sample was resuspended in 200 μL of ice-cold lysis buffer (0.5 M NaCl / 0.02 M Tris / 0.1% NP-40, pH 7) and sonicated for a minute on ice at a power of 180 W at an amplitude of 20% in pulses of 10 seconds sonication / 10 seconds of rest for each cycle (SONICA Q-500 Sonicator, Qsonica Llc., USA). For LC-ESI-MS/MS analysis, lipid extraction was achieved using the method of Folch, *et al.* [42]. Prior to extraction, samples were spiked with internal standards (40 pmol of C14 phosphatidylcholine (PC), 40 pmol of C17 phosphatidylethanolamine (PE), 15.8 pmol of d35GM1 ganglioside and 11.2 nmol of *d*_6_-cholesterol). Analysis of free cholesterol (FC), cholesteryl esters (CE), PC, PE and gangliosides was performed using a Shimadzu LC-20AD binary pump system combined with a AB Sciex API 4000 Q-trap triple-quadrupole mass spectrometer equipped with Analyst software (Version 1.4.2) and a turbo-ionspray source. Liquid chromatography separation was achieved by injecting samples (20 μL) onto a 3 μm Alltima C18 column (50 x 2.1 mm) at 200 μL/min using the following conditions; the HPLC gradient program began with 70% mobile phase A (30% tetrahydrofuran / 20% CH_3_OH / 50% 5 mM NH_4_COOH in H_2_O) followed by a linear ramp (0.01-7.0 min) to 100% mobile phase B (70% tetrahydrofuran / 20% CH_3_OH / 10% 5 mM NH_4_COOH in H_2_O) and maintained for 3 minutes. Re-equilibration at 70% mobile phase A was performed for 3 minutes prior to a subsequent injection. A Valco 10-port post column valve diverted column flow to waste for the first 1.7 minutes. Analysis of FC, CE, PC and sphingomyelin was performed in positive-ion multiple reaction monitoring mode using an ion spray temperature of 200°C and voltage of 5000 V. Mass spectrometric analysis of PE and gangliosides was performed in negative-ion multiple reaction monitoring mode using an ion spray temperature of 200°C and voltage of -5000 V. Nitrogen was used as the collision gas at a pressure of 2 x 10^-5^ Torr. Concentrations of each molecular species were calculated by relating the peak areas of each species to the peak area of the corresponding internal standard using Analyst 1.4.2 software.

The use of the *N*-acetylneuraminic acid fragment in the MRM pair for gangliosides negated the ability to differentiate the ceramide type, so we have denoted them with the total number of carbons and double bonds rather than two specified carbon chains. FC (and spiked *d*_6_-cholesterol internal standard) in each sample was converted to C2 cholesteryl ester by addition of 200 μL acetyl chloride/CHCl_3_ (1:5 v:v) and analysed by ESI-MS/MS as described by Liebisch *et al.* [43]. Relative cholesterol levels were determined by relating the peak area of C2 cholesterol to the peak area of the C2 *d*_6_-cholesterol internal standard. PC used the common product ion of *m*/*z* 184 corresponding to the phosphocholine head group and, therefore, PC species are denoted with the total number of carbons and double bonds rather than two specified carbon chains. The total protein level was used as loading control for the LC-ESI-MS/MS analysis, and the lipid content was presented as nmol mg^-1^ protein.

### FTIR spectroscopy

For FTIR spectroscopy cells were seeded at a density of 1 × 10^5^ cells mL^-1^ onto sterilised 2-mm thick CaF_2_ IR windows (Crystran, UK) using procedures developed previously for silicon nitride substrates [44]. After 24 hours fixed in 4% paraformaldehyde (PFA) for 30 minutes at room temperature and then dipped three times in double-distilled water. Slides with prostate cells were left to dry on the benchtop for 24 hours.

FTIR spectra maps were collected using a Bruker Vertex 80v FTIR spectrometer coupled to a Hyperion 3000 microscope, equipped with liquid-nitrogen-cooled 64 × 64 Focal Plane Array (FPA) detector. Instrument control and data collection was carried out using OPUS software (Version 7.0, Bruker, Ettlingen, Germany). Samples were continually purged with N_2_ to minimise water vapour and CO_2_ contributions in the spectral region 1350–1950 cm^-1^. FTIR images were collected using a ×36 microscope objective over the 3600–900 cm^-1^ spectral range with the co-addition of 1024 scans at a spectral resolution of 4 cm^-1^ from two individual cells for each cell line (*n* = 2). Analysis of a representative spectrum, generated from 14–20 spectral data points selected from two images and averaged from each cell line. A background spectrum was acquired from a blank CaF_2_ window before the collection of each sample image.

FTIR spectra were analysed using OPUS software. False-colour functional group images were generated by measuring the area under specific regions of interest in both the original and second derivatives of the spectral data. Second derivative of the spectra were generated in OPUS using the Savitsky-Golay smoothing function. FTIR images were generated to illustrate the lipid distribution and relative abundance within each cell line by integrating the area under the ν_s_(CH_2_) band (2862–2847 cm^−1^; Figure 2a^/^-2d^/^). The maximum intensity value of the FTIR image of 22RV1 cell had the highest integral intensity, therefore FTIR images from all cell lines were normalised against this value to allow direct comparison between the cell lines (Figure 2). For the selected spectral regions, PC-1 accounted for by far the greatest contribution to the spectral differences (98% for 3000–2800 cm^-1^ and 81% for 1750–1700 cm^-1^), and therefore was chosen for further data interrogation (Supplementary Figure 2a, 2c).

### Fluorescence imaging

For fluorescence imaging, prostate cells were seeded either at a density of 1 × 10^5^ cells mL^-1^ on #1.5 coverslips (*n* = 3 for each cell line) or 1 × 10^4^ cells mL^-1^ in 96-well plate (#CLS3603, Sigma-Aldrich, USA; *n* = 6 for each cell line), and were left to grow at 37°C with 5% CO_2_ for 24 and 48 hours, respectfully. Prior to staining with BODIPY^®^ 493/503 (1:100, #D3922, Life Technologies, USA) and Filipin III (1:1000, #F9765, Sigma-Aldrich, USA), cells were fixed with 4% PFA. ReZolve-L1™ (Rezolve Scientific, Australia) staining was performed on live cells (Figure 3i-3l) and 4% PFA fixed cells (Supplementary Figure 3a-23). Results in live cells yielded similar results to staining in fixed cells (Figure 3, Supplementary Figure 3); the intensity values of ReZolve-L1™ obtained in DU145, 22RV1 and LNCaP were higher than in PNT1a cells. Fixed cells were incubated with BODIPY^®^ 493/503, Filipin III or ReZolve-L1™ for 30 minutes according to manufactures guidelines. Live cells were incubated with 20 μM of ReZolve-L1™ in serum-free media for 30 minutes at 37°C and 5% CO_2_. Following staining, the cells were washed with sterile PBS and mounted for imaging.

Images were acquired with a Ziess LSM710 META NLO inverted microscope (Zeiss, Germany), which was supplemented with a two-photon Mai-Tai^®^, tunable Ti:Sapphire femtosecond pulse laser (710-920 nm, Spectra-Physics, USA). All imaging experiments were carried out at room temperature. Imaging BODIPY^®^ 493/503 was performed using argon-gas solid-state laser (Zeiss, Germany). Filipin III was detected using two-photon excitation wavelength 720 nm, beam splitter MBS 690+ and emission interval 407-480 nm. ReZolve-L1™ fluorescence was acquired at 820 nm two-photon excitation wavelength, beam splitter MBS 690+ and an emission interval of 493-601 nm. All images (*n* ≥ 10 for each cell line) were acquired using a Plan-APOCHROMAT 63X/ NA1.4 oil immersion objective. Each confocal micrograph represented 1.0 μm thin optical sections.

Prostate cells grown in 96-well plate, fixed in 4% PFA and stained with ReZolve-L1™ were imaged using Celldiscoverer 7 (Zeiss, Germany). ReZolve-L1™ fluorescence was acquired by utilising LED 385, with emission collected at 583-601 nm. Light source intensity was set to 10%, the depth of focus was 1.90 μm and the exposure time was 500 ms. For label-free imaging of cells, phase gradient contrast was employed. The number of images collected for each cell line was 30, and all of them were acquired using a Plan-APOCHROMAT 20X/ 0.7 dry objective.

### Statistical analysis

All data are presented as means ± s.e.m. Quantitative measurements of intensity values of ReZolve-L1™ staining in prostate cancer cell lines were made on digital images (*n* ≥ 10 for each cell line), using ZEN software (blue addition; Zeiss, Germany). The intensity value from each digital image (*n* ≥ 10) was plotted in GraphPad Prism (version 7 for Windows, GraphPad Software, San Diego, CA USA) for each designated group (PNT1a, DU145, 22RV1 and LNCaP). The statistically significant differences between group means were evaluated by one-way analysis of variance (ANOVA), with individual group variance assessed by a Bartlett's test. Where the level of significance was *p* < 0.05, post-hoc tests were performed using a Tukey's multiple comparison test. The fold change between PNT1a and cancer cell lines was obtained from the mean values (*n* = 6 for each lipid specie). Student’s *t*-test was used to assess the differences between PNT1a and prostate cell lines (i. e., DU145, 22RV1 and LNCaP) for the generation of the ‘volcano plot’. GraphPad Prism was used for all statistical analyses.

Principal component analysis (PCA) was performed using Unscrambler software (Version 9.7, CAMO, Olson, Norway). The multivariate statistical analysis method used in PCA allowed the data to be visualised in a reduced-dimension space, with each sample being converted into a single score [45]. PCA scores and loadings plots were obtained from the raw data. The score groups were plotted using selected principal components (PCs) as coordinates, with scores spatially separated based on sample similarity. The Hotelling’s T^2^ test, which corresponded to a multivariate generalisation of the 95% confidence interval, was utilised to identify outliers in the LC-ESI-MS/MS dataset.

### Image processing

Representative images and graphs were collated using Adobe Photoshop CC (Adobe Systems Inc, USA).

### Contributions

A.S. performed confocal imaging experiments, analysed LC-ESI-MS/MS and FTIR data by performing PCA and helped to prepare the manuscript. C.A.B. performed FTIR and confocal imaging experiments, aided in LC-ESI-MS/MS and FTIR data analysis and helped to prepare the manuscript. E.A.C. performed FTIR, aided PCA and provided intellectual contribution to FTIR analysis. I.R.D.J aided manuscript preparation and insight into biochemistry of prostate cancer. E.P. was involved in acquisition of LC-ESI-MS/MS analysis, which was performed at the SAHMRI. P.V.S. and P.J.W. synthesised and characterised ReZolve-L1™. S.S. was involved in design and synthesis of ReZolve-L1™. P.A.L contributed to FTIR analysis, insight into prostate cancer and manuscript preparation. M.M, D.A.B and S.E.P. helped to conceive experiments, prepare the manuscript, analysed the results, were involved in concept development, supervised the personnel and research direction and were responsible for acquiring project funding.

## SUPPLEMENTARY MATERIALS FIGURES AND TABLES




