# Supplementary material for: Lipid profiles of prostate cancer cells
Source: Oncotarget. 2018 Oct 30;9(85):35541–52. doi: 10.18632/oncotarget.26222 (PMC6238979; doi:10.18632/oncotarget.26222)
Supplement: Supplementary file 1 [file oncotarget-09-35541-s001.pdf]

Lipid profiles of prostate cancer cells

SUPPLEMENTARY MATERIALS

REFERENCES

1. Zhou X, Mao J, Ai J, Deng Y, Roth MR, Pound C, Henegar J, Welti R, Bigler SA. Identification of plasma lipid biomarkers for prostate cancer by lipidomics and bioinformatics. PLoS One. 2012; 7:e48889.
2. He M, Guo S, Li Z. *In situ* characterizing membrane lipid phenotype of breast cancer cells using mass spectrometry profiling. Sci Rep. 2015; 5:11298.
3. Ishikawa S, Tateya I, Hayasaka T, Masaki N, Takizawa Y, Ohno S, Kojima T, Kitani Y, Kitamura M, Hirano S, Setou M, Ito J. Increased expression of phosphatidylcholine (16:0/18:1) and (16:0/18:2) in thyroid papillary cancer. PLoS One. 2012; 7:e48873.
4. Cifkova E, Holcapek M, Lisa M, Vrana D, Melichar B, Student V. Lipidomic differentiation between human kidney tumors and surrounding normal tissues using HILIC-HPLC/ESI-MS and multivariate data analysis. J Chromatogr B Analyt Technol Biomed Life Sci. 2015; 1000:14-21.
5. Llorente A, Skotland T, Sylvanne T, Kauhanen D, Rog T, Orlowski A, Vattulainen I, Ekroos K, Sandvig K. Molecular lipidomics of exosomes released by PC-3 prostate cancer cells. Biochim Biophys Acta. 2013; 1831:1302-1309.
6. Hilvo M, Denkert C, Lehtinen L, Muller B, Brockmoller S, Seppanen-Laakso T, Budczies J, Bucher E, Yetukuri L, Castillo S, Berg E, Nygren H, Sysi-Aho M, Griffin JL, Fiehn O, Loibl S, et al. Novel theranostic opportunities offered by characterization of altered membrane lipid metabolism in breast cancer progression. Cancer Res. 2011; 71:3236-3245.
7. Zhuang L, Kim J, Adam RM, Solomon KR, Freeman MR. Cholesterol targeting alters lipid raft composition and cell survival in prostate cancer cells and xenografts. J Clin Invest. 2005; 115:959-968.
8. Guo S, Qiu L, Wang Y, Qin X, Liu H, He M, Zhang Y, Li Z, Chen X. Tissue imaging and serum lipidomic profiling for screening potential biomarkers of thyroid tumors by matrix-assisted laser desorption/ionization-Fourier transform ion cyclotron resonance mass spectrometry. Anal Bioanal Chem. 2014; 406:4357-4370.
9. Yoshimura K, Chen LC, Mandal MK, Nakazawa T, Yu Z, Uchiyama T, Hori H, Tanabe K, Kubota T, Fujii H, Katoh R, Hiraoka K, Takeda S. Analysis of renal cell carcinoma as a first step for developing mass spectrometry-based diagnostics. J Am Soc Mass Spectrom. 2012; 23:1741-1749.
10. Baker MJ, Trevisan J, Bassan P, Bhargava R, Butler HJ, Dorling KM, Fielden PR, Fogarty SW, Fullwood NJ, Heys KA, Hughes C, Lasch P, Martin-Hirsch PL, Obinaju B, Sockalingum GD, Sule-Suso J, et al. Using Fourier transform IR spectroscopy to analyze biological materials. Nat Protoc. 2014; 9:1771-1791.
11. Fagone P, Jackowski S. Membrane phospholipid synthesis and endoplasmic reticulum function. Journal of lipid research. 2009; 50:S311-316.
12. Fu S, Watkins SM, Hotamisligil GS. The role of endoplasmic reticulum in hepatic lipid homeostasis and stress signaling. Cell Metab. 2012; 15:623-634.
13. Hackett MJ, Lee J, El-Assaad F, McQuillan JA, Carter EA, Grau GE, Hunt NH, Lay PA. FTIR imaging of brain tissue reveals crystalline creatine deposits are an *ex vivo* marker of localized ischemia during murine cerebral malaria: general implications for disease neurochemistry. ACS Chem Neurosci. 2012; 3:1017-1024.
14. Coates J. (2006). Interpretation of Infrared Spectra, A Practical Approach. Encyclopedia of Analytical Chemistry: John Wiley & Sons, Ltd.
15. Movasaghi Z, Rehman S, ur Rehman DI. Fourier Transform Infrared (FTIR) Spectroscopy of Biological Tissues. Applied Spectroscopy Reviews. 2008; 43:134-179.

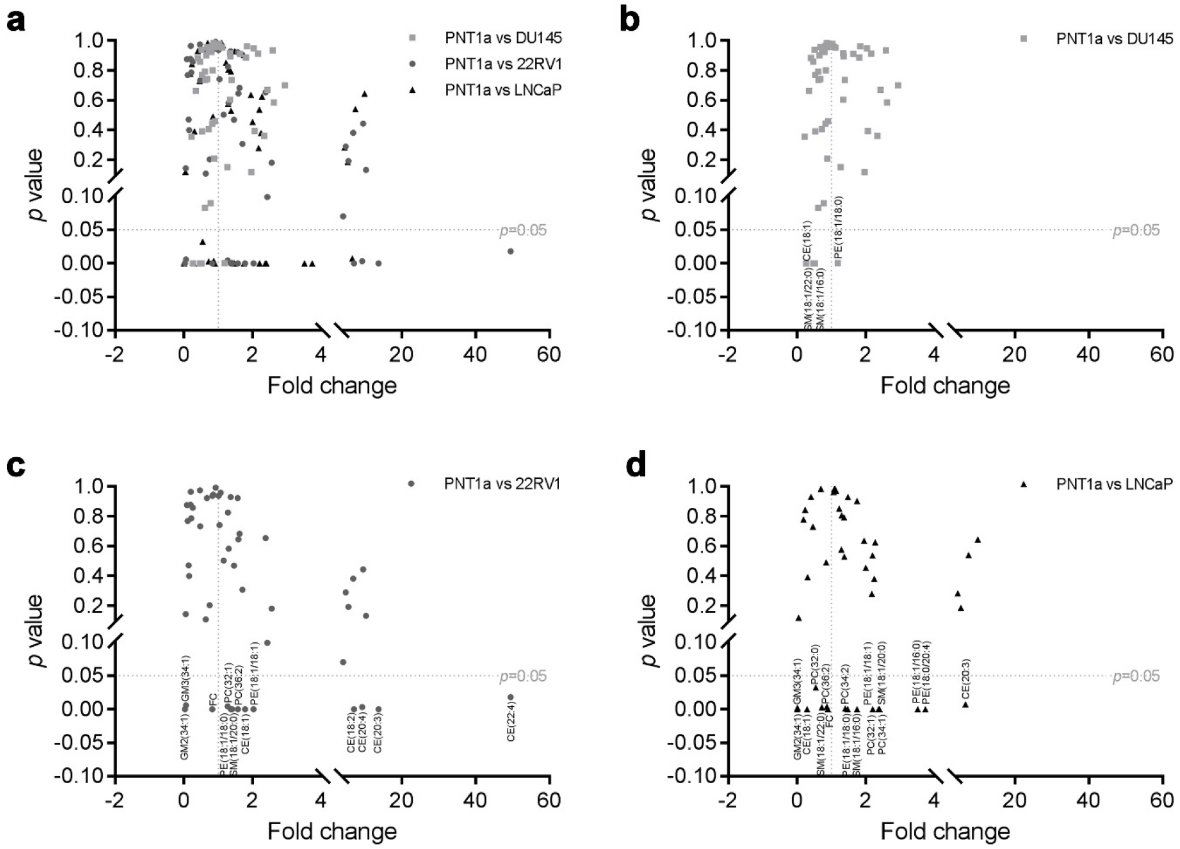

Supplementary Figure 1: Volcanos plot showing differences in lipid composition between PNT1a and prostate cancer cell lines. (a) Illustrates comparison between PNT1a and all three prostate cancer cell lines. **(b-c)** Shows comparison between PNT1a and individual prostate cancer cell lines as indicated. The *x*-axis shows fold change relative to PNT1a, where 1 indicated no difference between cell lines. The *y*-axis shows $\log_{10}$ adjusted *p* values. The dashed horisontal line represents statistical significance threshold ($p < 0.05$). n = 6 for each cell line.

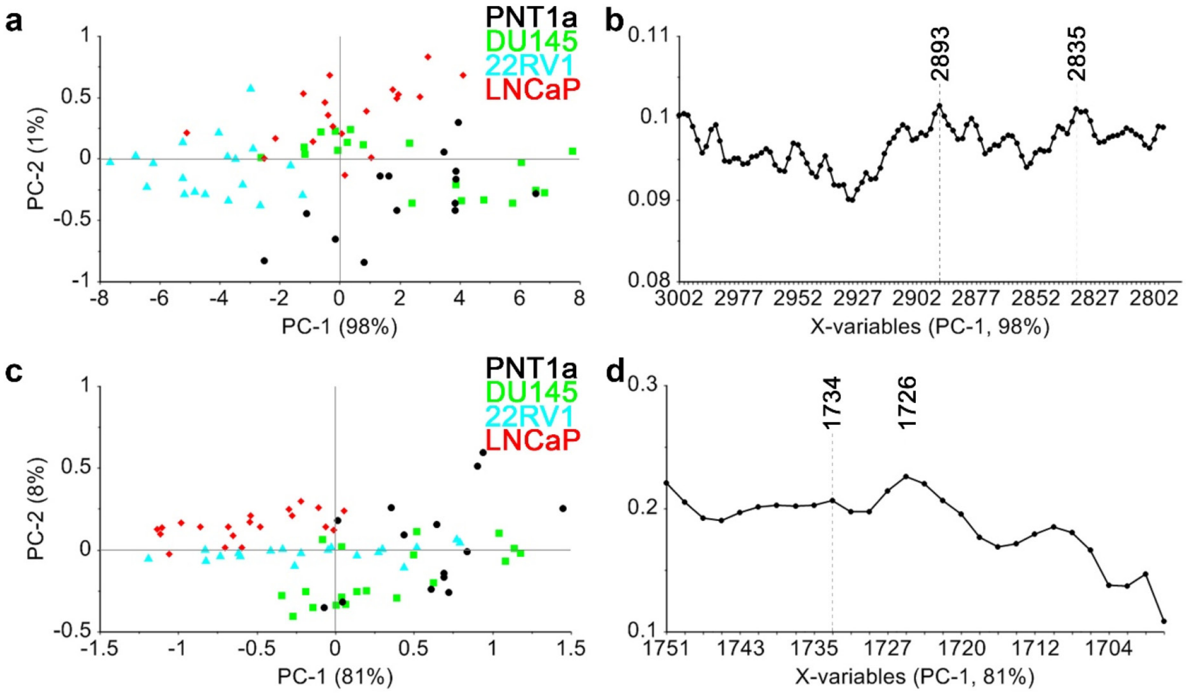

Supplementary Figure 2: PCA showing the variability of the C-H and C=O stretching regions between PNT1a and prostate cancer cells. (a, c) PCA scores plots comparing PNT1a (black circles) and prostate cancer cell lines DU145 (green squares), 22RV1 (blue triangles) and LNCaP (red diamonds), using 3000–2800 cm^{-1}, (a) or 1750-1700 cm^{-1} spectral regions (c). **(b, d)** PCA loadings plots obtained for PC-1, which contributed to (b) 98% and (d) 81% variability.

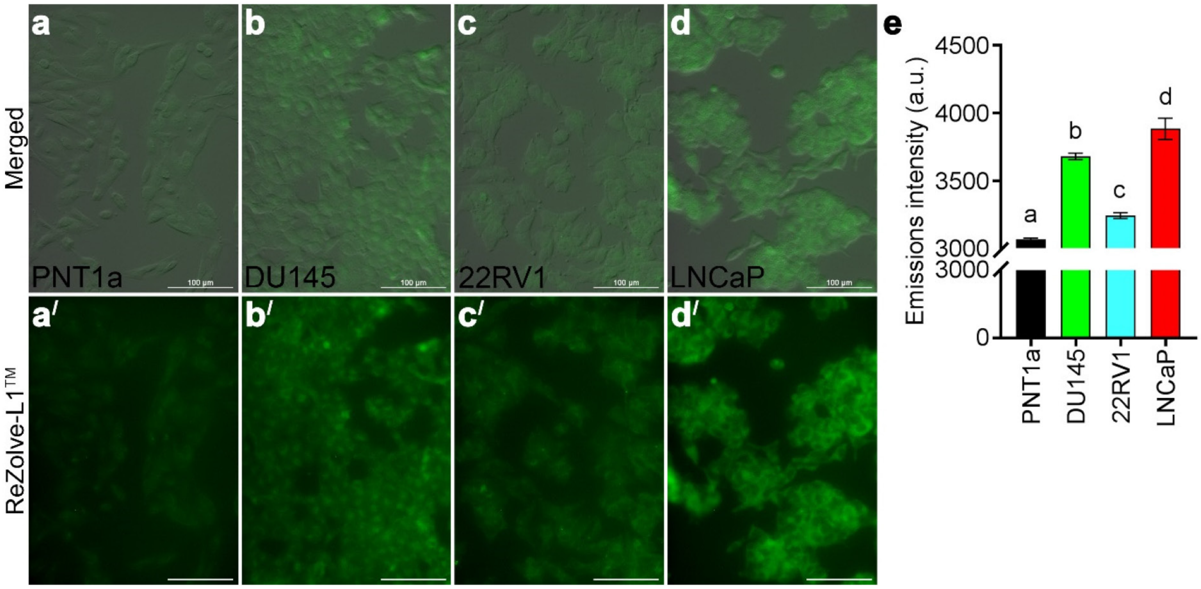

Supplementary Figure 3: Phosphorescence intensity of ReZolve-L1™ in prostate cell lines. (a-d) Representative micrographs showing PNT1a and prostate cancer DU145, 22RV1 and LNCaP cells (a-d; phase gradient contrast) stained with ReZolve-L1™ (a′-d′). Prostate cells were fixed with 4% PFA. Scale bars, 100 µm. **(e)** Histogram showing comparative analysis of ReZolve-L1™ phosphorescence intensity in prostate cell lines. One-way ANOVA and Tukey's multiple comparison test showed significant differences between the means in designated groups (depicted by different letters on the bars, $p < 0.05$).

Supplementary Table 1: Lipid species in differentiation of prostate cancer

See Supplementary File 1

Supplementary Table 2: Position and assignment of FTIR lipid bands of interest found in prostate cells

Cell line	ν(CH)	$\nu_{as}(CH_3)$	$\nu_s(CH_3)$	$\nu_{as}(CH_2)$	$\nu_s(CH_2)$	ν(C=O)
PNT1a	2900 2832	2959	2877	2928	2854	1738
DU145	2896 2828	2959	2875	2923	2851	1744
22RV1	2897 2826	2959	2875	2924	2852	1745
LNCaP	2898 2827	2959	2875	2923	2852	1738

The band assignments and wavenumber data (cm^{-1}) were obtained from the analysis of second derivatives of the lipid spectra. ν – stretching, as – antisymmetric, s – symmetric.

Supplementary Table 3: Position and Assignment of IR lipids bands of interest found in prostate cells

Lipid	ν(CH)	$\nu_{as}(CH_3)$	$\nu_s(CH_3)$	$\nu_{as}(CH_2)$	$\nu_s(CH_2)$	ν(C=O)
Phosphatidylethanolamine		2959		2922	2852	1741
Phosphatidylcholine		2959		2922	2852	1739
Sphingomyelin		2961	2876	2921	2852	
Sphingosine	2990	2957	2876	2917	2849	
Cholesterol	2900	2962	2867	2932	2846	
Cholesteryl acetate	2908, 2824	2968		2939	2861	1732
Cholesteryl behenate	2892	2957	2871	2917	2850	1739
Fatty acid		2958	2876	2918	2850	
Triacylglycerides		2960	2875	2928	2855	1745

The data were obtained from the analysis of second derivatives of the lipid spectra.
ν – stretching, as – antisymmetric, s – symmetric.
IR lipids bands reviewed in [14, 15].
