# Supplementary material for: Lipid profiles of prostate cancer cells
Source: Oncotarget. 2018 Oct 30;9(85):35541–52. doi: 10.18632/oncotarget.26222 (PMC6238979; doi:10.18632/oncotarget.26222)
Supplement: Supplementary file 2 [file oncotarget-09-35541-s002.docx]

| Table S1. Lipid species in differentiation of prostate cancer. | | | | | | | | |  |
| --- | --- | --- | --- | --- | --- | --- | --- | --- | --- |
| Lipids | **PNT1a** | **DU145** | | **22RV1** | | **LNCaP** | | **Reported up- or down-regulation in cancer** |  |
|  | **Mean ± SEM** | **Mean ± SEM** | **Relative change** | **Mean ± SEM** | **Relative change** | **Mean ± SEM** | **Relative change** |  |  |
| CE (16:2) | 18.40 ± 1.94 | 9.47 ± 0.95 | - 1.9 | 124.80 ± 4.83 | + 6.8 | 32.20 ± 2.65 | + 1.8 |  | |
| CE (16:1) | 140.60 ± 9.06 | 31.57 ± 1.49 | - 4.5 | 341.20 ± 13.95 | + 2.4 | 42.97 ± 1.72 | - 3.3 |  | |
| CE (16:0) | 72.98 ± 4.17 | 38.70 ± 1.07 | - 1.9 | 293.30 ± 18.81 | + 4.0 | 33.67 ± 0.69 | - 2.2 |  | |
| CE (18:3) | 19.88 ± 1.83 | 13.58 ± 0.30 | - 1.5 | 203.30 ± 5.70 | + 10.2 | 21.92 ± 0.84 | + 1.1 |  | |
| CE (18:2) | 81.24 ± 4.21 | 50.38 ± 1.54 | - 1.6 | 569.30 ± 22.26 | + 7.0 | 111 ± 3.75 | + 1.4 | Up-regulated in plasma from prostate cancer patients [1]. | |
| CE (18:1) | 1052 ± 99.03 | 276.80 ± 9.00 | - 3.8 | 1879 ± 116.20 | + 1.8 | 306.20 ± 8.48 | - 3.4 |  | |
| CE (18:0) | 39.50 ± 1.42 | 18.58 ± 0.55 | - 2.1 | 93.95 ± 4.52 | + 2.4 | 7.57 ± 0.20 | - 5.2 | Up-regulated in plasma from prostate cancer patients [1]. | |
| CE (20:5) | 34.54 ± 6.48 | 25.12 ± 1.25 | - 1.4 | 163.30 ± 5.26 | + 4.7 | 37.27 ± 1.70 | + 1.1 |  | |
| CE (20:4) | 43.70 ± 3.83 | 59.17 ± 0.98 | + 1.4 | 401.70 ± 10.65 | + 9.2 | 99.48 ± 4.41 | + 2.3 |  | |
| CE (20:3) | 55.32 ± 3.03 | 62.27 ± 1.84 | + 1.1 | 754.80 ± 27.17 | + 13.6 | 362 ± 11.05 | + 6.5 |  | |
| CE (22:6) | 35.22 ± 3.22 | 85.48 ± 3.09 | + 2.4 | 193.80 ± 3.86 | + 5.5 | 186.50 ± 7.63 | + 5.3 |  | |
| CE (22:5) | 10.99 ± 0.66 | 23.80 ± 0.66 | + 2.2 | 104.40 ± 7.94 | + 9.5 | 80.85 ± 4.23 | + 7.4 | Up-regulated in plasma from prostate cancer patients [1]. | |
| CE (22:4) | 5.95 ± 0.47 | 15.37 ± 0.54 | + 2.6 | 294.30 ± 11.49 | + 49.5 | 58.50 ± 1.66 | + 9.8 |  | |
| CE (24:1) | 16.78 ± 1.33 | 14.57 ± 0.41 | - 1.2 | 15.60 ± 0.69 | - 1.1 | 6.82 ± 0.43 | - 2.5 |  | |
| SM (18:1/20:0) | 1972 ± 46.96 | 902.50 ± 12.84 | - 2.2 | 2698 ± 53.71 | + 1.4 | 4734 ± 127 | + 2.4 |  | |
| SM (18:1/16:0) | 979 ± 23.70 | 510.20± 6.37 | - 1.9 | 1017 ± 13.83 | + 1.0 | 1713 ± 56.53 | + 1.7 | Up-regulated in breast cancer SK-BR-3 and MDA-MB-361 cell lines [2], and thyroid papillary cancer [3]. | |
| SM (18:1/16:1) | 75.52 ± 1.96 | 26.65 ± 0.40 | - 2.8 | 36.05 ± 0.70 | - 2.1 | 80.53 ± 1.54 | + 1.1 |  | |
| SM (18:1/18:0) | 99.77 ± 2.22 | 61.88 ± 0.54 | - 1.6 | 255 ± 3.11 | + 2.6 | 217.30 ± 4.40 | + 2.2 | Down-regulated in kidney tumours [4]. | |
| SM (18:1/18:1) | 27.82 ± 0.63 | 11.37 ± 0.31 | - 2.4 | 38.05 ± 0.38 | + 1.4 | 31.77 ± 0.73 | + 1.1 | Down-regulated in kidney tumours [4]. | |
| SM (18:1/22:0) | 1159 ± 21.60 | 547.50 ± 7.02 | - 2.1 | 1824 ± 110.70 | + 1.6 | 834.30 ± 16.27 | - 1.4 | Enriched in PC-3 exosomes [5]. | |
| SM (18:1/24:0) | 206.70 ± 4.05 | 110.30 ± 1.52 | - 1.9 | 270.30 ± 5.10 | + 1.3 | 267.30 ± 10.59 | + 1.3 | Up-regulated in breast cancer [6]; enriched in PC-3 exosomes [5]. | |
| SM (18:1/24:1) | 491.80 ± 10.68 | 405.50 ± 3.77 | - 1.2 | 569.50 ± 4.40 | + 1.2 | 416.80 ± 8.59 | - 1.2 |  | |
| Cholesterol | 15900 ± 524.70 | 17683 ± 574.10 | + 1.1 | 13133 ± 497.10 | -1.2 | 14267 ± 374.80 | - 1.1 | Enriched in PC-3 exosomes [5]; the survival of prostate cancer cells is dependent on a cholesterol-regulated Akt pathway [7]. | |
| PE (16:0/22:4) | 34.03 ± 1.29 | 45.80 ± 0.95 | + 1.3 | 22.92 ± 1.80 | - 1.5 | 151 ± 3.18 | + 4.4 | Up-regulated in breast cancer BT-20 cell line [2]. | |
| PE (18:0/18:0) | 88.55 ± 3.24 | 91.62 ± 1.96 | 1.0 | 114 ± 2.72 | + 1.3 | 115.20 ± 1.49 | + 1.3 |  | |
| PE (18:0/18:2) | 53.98 ± 2.51 | 43.23 ± 0.67 | - 1.2 | 46.18 ± 1.80 | - 1.2 | 105.50 ± 1.89 | + 2.0 | Expression levels are associated with the types of breast cancer cell lines [2]; up-regulated in plasma from prostate cancer patients [1] and kidney tumours [4]. | |
| PE (18:0/20:4) | 168.20 ± 7.16 | 226.70 ± 5.10 | + 1.3 | 286.50 ± 17.86 | + 1.7 | 628.30 ± 11.20 | + 3.7 | Up-regulated in grade 3 tumours of breast cancer [6] and BT-20 cell line [2]; down-regulated in breast cancer MCF-7 cell line [2] and kidney tumours [4]. | |
| PE (18:0/22:6) | 19.78 ± 1.04 | 35.83 ± 0.82 | + 1.8 | 31 ± 3.35 | + 1.6 | 29.35 ± 1.02 | + 1.5 | Up-regulated in kidney tumours [4]. | |
| PE (18:1/16:0) | 598.50 ± 16.87 | 760.30 ± 19.77 | + 1.3 | 450.80 ± 17.06 | - 1.3 | 2103 ± 20.16 | + 3.5 | Down-regulated in kidney tumours [4]. | |
| PE (18:1/16:1) | 183.20 ± 5.48 | 359.70 ± 4.98 | + 2.0 | 267 ± 10.47 | + 1.5 | 251.50 ± 8.17 | + 1.4 | Down-regulated in kidney tumours [4]. | |
| PE (18:1/18:0) | 2106 ± 76.58 | 2499 ± 45.06 | + 1.2 | 2847 ± 83.25 | + 1.4 | 3048. ± 58.07 | + 1.4 | Up-regulated in kidney tumours [4] and grade 3 tumours of breast cancer [6]. | |
| PE (18:1/18:1) | 2140 ± 65.55 | 3545 ± 73.73 | + 1.7 | 4347 ± 157.60 | + 2.0 | 3149 ± 37.63 | + 1.5 | Enriched in PC-3 exosomes [5]; up-regulated in plasma from prostate cancer patients [1]. | |
| PE (18:1/18:2) | 90.58 ± 2.79 | 186.80 ± 3.61 | + 2.1 | 143.80 ± 3.03 | + 1.6 | 111 ± 3.84 | + 1.2 | Down-regulated in kidney tumours [4]. | |
| PE (18:1/20:4) | 76.60 ± 3.26 | 179.30 ± 3.04 | + 2.3 | 124 ± 3.23 | + 1.6 | 172.20 ± 3.13 | + 2.2 | Down-regulated in kidney tumours [4]. | |
| PC (32:0) | 514.50 ± 10.87 | 319.20 ± 4.07 | - 1.6 | 328 ± 20.20 | - 1.6 | 281.80 ± 12.93 | - 1.8 | Enriched in PC-3 exosomes [5]; expression levels are associated with the types of breast cancer cell lines [2]; up-regulated in grade 3 tumours of breast cancer [6], kidney tumours [4] and malignant thyroid cancer [8]. | |
| PC (32:1) | 1193 ±22.24 | 1051 ± 12.65 | - 1.1 | 1524 ± 13.31 | + 1.3 | 2633 ± 32.83 | + 2.2 | Up-regulated in breast cancer, BT-20, MCF-7, SK-BR-3 and MDA-MB-231, cell lines [2], grade 3 tumours of breast cancer [6]. | |
| PC (34:1) | 5077 ± 100 | 2367 ± 26.82 | - 2.1 | 7120 ± 124.50 | + 1.4 | 12045 ± 293.20 | + 2.4 | Up-regulated in plasma from prostate cancer patients [1], thyroid papillary cancer [3] and malignant thyroid cancer [8]; expression levels are associated with the types of breast cancer cell lines [2]. | |
| PC (34:2) | 876.30 ± 18.22 | 685.30 ± 12.38 | - 1.3 | 885.30 ± 15.65 | 1.0 | 1233 ± 24.08 | + 1.4 | Up-regulated in plasma from prostate cancer patients [1] and thyroid papillary cancer [3]; down-regulated in kidney tumours [4]. | |
| PC (36:2) | 2478 ± 37.65 | 1367 ± 17.05 | - 1.8 | 3851 ± 232.80 | + 1.6 | 2161 ± 51.07 | - 1.1 | Up-regulated in breast cancer MDA-MB-231 cell line [2], thyroid tumours [8] and kidney tumours [9]. | |
| PC (36:4) | 56.12 ± 0.76 | 50.17 ± 0.54 | - 1.1 | 46.93 ± 3.26 | - 1.2 | 123.20 ± 3.16 | + 2.2 | Down-regulated in thyroid tumours [8] and kidney tumours [9]. | |
| PC (38:4) | 81.42 ± 1.59 | 87.18 ± 1.28 | + 1.1 | 87.42 ± 6.64 | + 1.1 | 162.80 ± 4.16 | + 2.0 | Down-regulated in breast cancer, BT-20, MCF-7, SK-BR-3, MDA-MB-231, MDA-MB-157 and MDA-MB-361, cell lines [2]; up-regulated in plasma from prostate cancer patients [1], malignant kidney tumours [4] and thyroid tumours [8]. | |
| GM1 (34:1) | 179 ± 8.72 | 150 ± 6.62 | - 1.2 | 9.37 ± 2.21 | - 19.1 | 8.33 ± 0.49 | - 21.5 |  | |
| GM1 (36:1) | 28.15 ± 1.48 | 23.25 ± 1.64 | - 1.2 | 7.37 ± 1.06 | - 3.8 | 8.81 ± 0.36 | - 4.8 |  | |
| GM1 (38:1) | 6.89 ± 0.90 | 14.03 ± 1.44 | + 2.0 | 3.26 ± 0.24 | - 2.1 | 4.94 ± 0.83 | -1.4 |  | |
| GM2 (34:1) | 848.60 ± 42.59 | 765.50 ± 35.13 | - 1.1 | 31.72 ± 2.42 | - 26.8 | 6.72 ± 0.51 | - 126.2 |  | |
| GM2 (36:1) | 115 ± 3.37 | 78.07 ± 2.77 | - 1.5 | 17.40 ± 1.87 | - 6.6 | BD |  |  | |
| GM2 (38:1) | 19.85 ± 1.41 | 32.43 ± 2.19 | + 1.6 | 1.83 ± 0.43 | - 10.8 | BD |  |  | |
| GM3 (34:1) | 340.70± 13.72 | 247 ± 17.38 | - 1.4 | 20.75 ± 1.82 | - 16.4 | 4.62 ± 0.83 | - 73.7 |  | |
| GM3 (36:1) | 39.98 ± 2.74 | 26.17 ± 1.46 | - 1.5 | 8.62 ± 0.50 | - 4.6 | BD |  |  | |
| GM3 (38:1) | 6.31 ± 0.54 | 11.59 ± 1.46 | + 1.8 | 1.28 ± 0.48 | - 4.9 | BD |  |  | |
| GM3 (40:1) | 22.23 ± 1.01 | 65.45 ± 4.80 | + 2.9 | 4.29 ± 0.53 | - 5.2 | BD |  |  | |
| GM3 (42:2 | 97.12 ± 5.34 | 135 ± 14.59 | + 1.4 | 13.64 ± 1.82 | - 7.1 | BD |  |  | |
| GM3 (42:1) | 38.13 ± 1.91 | 99.78 ± 11.67 | + 2.6 | 4.14 ± 0.37 | - 9.2 | BD |  |  | |
| Lipid species identities were based on the mass/charge ration of the intact lipid ion.  Lipid classes: CE – cholesteryl esters; SM – sphingomyelin; PE – phosphatidylethanolamine; PC – phosphatidylcholine.; GM – ganglioside. Concentration: pmol mg^-1^ protein.  Relative change in lipid concentration is calculate as the fold increase (+) or decrease (-) of the average lipid concentration in malignant cell lines compared to non-malignant control, PNT1a, average values. A value of│1│ indicated not change in the average lipid concentration.  BD = Below the Detection level. | | | | | | | | | |
|  | | | | | | | | | |
